# Ketogenic dietary therapies in epilepsy: recommendations of the Italian League against Epilepsy Dietary Therapy Study Group

**DOI:** 10.3389/fneur.2023.1215618

**Published:** 2023-07-10

**Authors:** Valentina De Giorgis, Anna Tagliabue, Francesca Bisulli, Ilaria Brambilla, Alessandra Camerini, Raffaella Cusmai, Francesca Darra, Alice Dianin, Elia Domenica, Monica Anna Maria Lodi, Sara Matricardi, Tullio Messana, Francesca Operto, Francesca Ragona, Emilio Russo, Costanza Varesio, Lilia Volpi, Martina Paola Zanaboni, Ludovica Pasca, Pierangelo Veggiotti

**Affiliations:** ^1^Department of Brain and Behaviour Neuroscience, University of Pavia, Pavia, Italy; ^2^Child Neurology and Psychiatry Unit, IRCCS Mondino Foundation, Pavia, Italy; ^3^Department of Public Health, Human Nutrition and Eating Disorder Research Center and Ketogenic Metabolic Therapy Laboratory—Experimental and Forensic Medicine University of Pavia, Pavia, Italy; ^4^IRCCS Istituto delle Scienze Neurologiche di Bologna, Full Member of the European Reference Network for Rare and Complex Epilepsies (EpiCARE), Bologna, Italy; ^5^Department of Biomedical and Neuromotor Sciences, University of Bologna, Bologna, Italy; ^6^Endocrinologia, Diabetologia e Ginecologia Pediatrica, Fondazione IRCCS Policlinico San Matteo di Pavia, Università degli Studi di Pavia, Pavia, Italy; ^7^Italian GLUT1-DS Association, Milan, Italy; ^8^Child Neurology Unit, Department of Neuroscience and Neurorehabilitation, Bambino Gesù Children's Research Hospital, IRCCS, Rome, Italy; ^9^Child Neuropsychiatry Unit, Department of Engineering for Innovation Medicine, University of Verona, Full Member of European Reference Network EpiCARE, Verona, Italy; ^10^Inherited Metabolic Diseases Unit and Regional Centre for Newborn Screening, Diagnosis and Treatment of Inherited Metabolic Diseases and Congenital Endocrine Diseases, Azienda Ospedaliera Universitaria Integrata, Verona, Italy; ^11^Artificial Nutrition Unit, Bambino Gesù Children's Hospital IRCCS, Rome, Italy; ^12^Department of Child Neuropsychiatry, Epilepsy Center, Fatebenefratelli Hospital, Milan, Italy; ^13^Department of Pediatrics, University of Chieti, Chieti, Italy; ^14^Istituto delle Scienze Neurologiche di Bologna, UOC Neuropsichiatria dell'etá pediatrica, Member of the ERN Epicare, Bologna, Italy; ^15^Department of Science of Health, School of Medicine, University Magna Graecia of Catanzaro, Catanzaro, Italy; ^16^Department of Pediatric Neurology, IRCCS Foundation Carlo Besta Neurological Institute, Milan, Italy; ^17^Science of Health Department, University of Catanzaro, Catanzaro, Italy; ^18^UOC Neurologia, IRCCS Istituto delle Scienze Neurologiche, Azienda USL di Bologna, Ospedale Bellaria Bologna, Bologna, Italy; ^19^Vittore Buzzi Children's Hospital, Pediatric Neurology Unit, Milan, Italy; ^20^Department of Biomedical and Clinical Sciences, L. Sacco, University of Milan, Milan, Italy

**Keywords:** Ketogenic dietary therapies, consensus, recommendations, drug resistant epilepsy, multidisciplinary approach, target treatment

## Abstract

A stepwise increase in the utilization of ketogenic dietary therapies for drug-resistant epilepsy has been observed in Italy in the last decade, although it is still considered often underused in many centers when compared to other countries. The Dietary Therapy Study Group of the Italian League against Epilepsy proposes practical recommendations to improve shared knowledge and facilitate the application of ketogenic dietary therapies, optimizing its efficacy and tolerability. The experts involved (11 child neuropsychiatrists, two adult neurologists, one psychologist, one pharmacologist, one pediatric endocrinologist, one representative of patients' associations, and three dietitians and clinical nutritionists) responded to a survey on current clinical practice issues and were asked to discuss controversial topics related to supplementation, long-term maintenance, transition, and a multidisciplinary approach to ketogenic dietary therapies. Practical indications for patient selection, diet initiation, management, side effects prevention, and follow-up are provided.

## 1. Background

Ketogenic dietary therapies (KDTs) have been widely used to treat epilepsy since the 1920s−1930's (1), but their use significantly decreased with the discovery of new anti-seizure medications (ASMs). However, KDTs regained popularity in the 1990s, with promising research published in those years (2–5). In contemporary times, KDTs are increasingly accepted worldwide as a practical, effective, and safe alternative treatment for children and adults with refractory epilepsy who are not good surgical candidates. Moreover, to date, several types of KDT are available to improve efficacy and compliance (6). Until the initial years of this century, Italian epilepsy centers with an available KDT service were few. In the last few years, KDTs have become more widespread across the country owing to improved knowledge and education.

This manuscript relies on a consensus elaborated within the Dietary Therapy Study Group of the Italian League against Epilepsy (LICE) to ameliorate and facilitate the application of KDTs and expand their use. These recommendations have been mainly intended for Italian neurologists, neuropsychiatrists, and dieticians, considering the Italian health system and social and dietary habits of the Italian population. The authors also attempted to address some universally shared gaps in KDT provision and management in the long term, thus making these recommendations of interest for all KDT services, regardless of their geographical location.

Members of the Dietary Taskforce are distributed throughout the Italian territory, thus contributing to the regional specificities of the Italian health system. The group of 19 authors comprised 11 child neuropsychiatrists, two adult neurologists, one psychologist, one pharmacologist, one pediatric endocrinologist, one representative of the Patients' Association, and three dietitians, as well as clinical nutritionists. This consensus was then circulated to all authors for comments and revisions. Surveys were circulated regarding controversial issues, and a final face-to-face consensus meeting was held during the Workshop “GdS dietoterapie LICE – La relazione tra credenze, conoscenze e aderenza alla dieta chetogenica: prospettive a confronto” organized on 12–13 May 2022 in Postal (Italy) in a dedicated section. Authors were instructed to cite peer-reviewed publications whenever available. In the absence of published literature, the participants were asked to base their recommendations on their professional or collective medical center experience. The results of the survey on “new” or controversial topics and informative data on the current practice across the national territory were then incorporated into the body of the manuscript, providing percentage responses for topics. Sections were collected, merged into a whole document, and then emailed to the entire group for review. All participants reviewed and approved the final manuscript before submission.

## 2. Different types of diet: a matter of definitions

A ketogenic diet (KD) usually refers to any dietary therapy in which dietary composition would be expected to result in a ketogenic state of human metabolism. Ketosis is the result of starvation or semi-starvation, with the latter (i.e., a very low-calorie ketogenic diet, VLCKD) being used for weight loss purposes in obesity and other metabolic diseases (7). In contrast, ketosis is induced in patients with neurological diseases by using a full-calorie dietary regimen with very high fat, adequate protein, and very low carbohydrate content. As such, KD provides ample lipids, which are processed into free fatty acids in the liver and then oxidized in mitochondria, producing high levels of acetyl-coenzyme A, which is partially converted into ketone bodies. The term KD has recently been substituted by KDTs because several dietary protocols are used for this purpose. The dietary protocols differ in ketogenic ratios and food choices. The classic ketogenic diet (cKD) implies the strictest dietary protocols (mainly 3:1 or 4:1 fat to protein plus carbohydrate ratio) in which each ingredient needs to be weighted. It may contain either long-chain fatty acids and/or medium-chain fatty acids. Some families and patients find adherence to the cKD difficult. Therefore, other more liberal versions, such as the medium-chain triglycerides KD (MCT-KD), modified Atkins diet (MAD), and low glycemic index treatment (LGIT), have been proposed.

Each dietary therapy has advantages and disadvantages, i.e., the classical protocol reaches the maximum level of blood ketosis but might induce long-term side effects due to compensated acidosis, which is a hallmark of the therapy. Better compliance and avoidance of long-term side effects can be obtained by a careful baseline patient evaluation, focusing on energy needs and body composition, individual and family dietary habits, food intolerances, and/or comorbidities that require special diets. In clinical practice, many patients with severe encephalopathies use artificial nutrition, particularly percutaneous endoscopic gastrostomy (PEG) nutrition. Ketogenic protocols may be administered and can be prepared either with natural foods or with formula. Some patients have coeliac disease, which requires avoiding gluten. Gluten is contained in some cereals, which are represented in minor amounts in this very low-carb regimen. Moreover, meals can be prepared with gluten-free products. On the contrary, milk and dairy products are always included in KDTs and are a significant source of lactose, and this might represent an issue for lactose-intolerant patients. However, the amount varies between food sources, and milk can be substituted with lactose-free milk or yogurt. An expert keto team should manage each situation.

**Figure 1:**
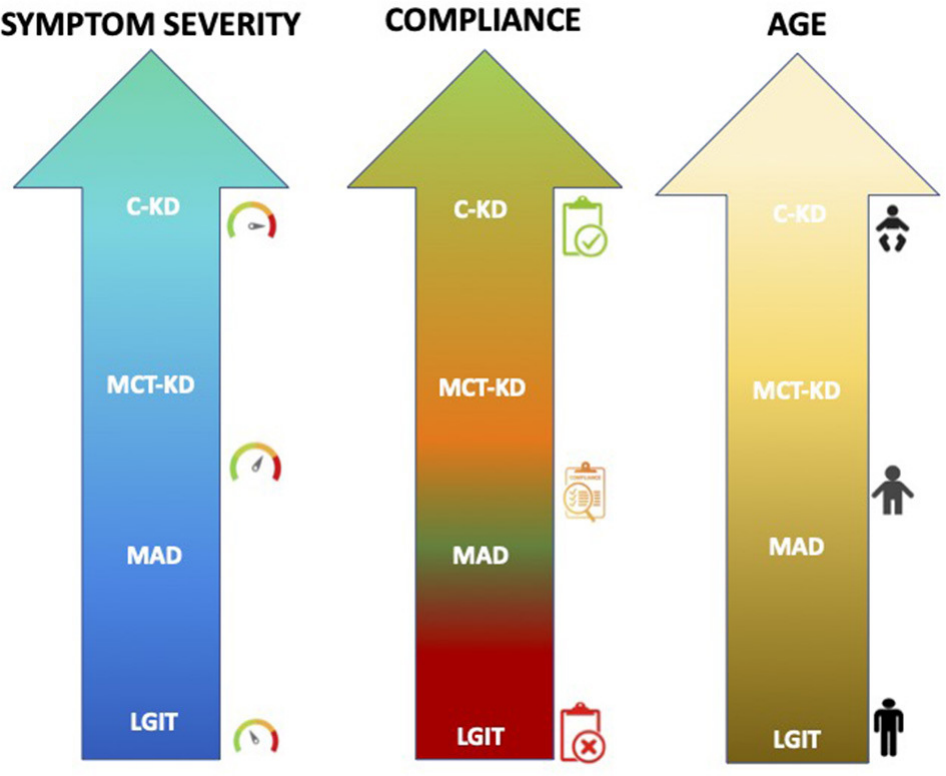
The most common dietary therapies prescribed in relation to the age and clinical condition of the patients among our Italian referral centers. A personalized approach to the elaboration of the diet is, however, the common element of all the approaches.

The dietary protocol most prescribed among the consensus members is cKD (60%), followed by MAD (25%), primarily used in adolescent-adult patients, and MCT-KD (10%), used in patients with compliance problems, while LGIT is prescribed in ~5% (Figure 1).

## 3. Patient selection

In the international consensus study group (6), it has been stated that KDTs should be offered to a child after a mean of 2.6 (standard deviation; SD 0.9) during which anti-seizure drugs were tested unsuccessfully. In particular, several conditions for which KDTs have been reported to be greatly beneficial (>70%) than the average 50% KDT response have been outlined: developmental and epileptic encephalopathies (DEE), GLUT1 deficiency syndrome (GLUT1DS), pyruvate dehydrogenase deficiency (PDH), Angelman syndrome, and tuberous sclerosis complex (6). In those conditions, the international consensus study group recommends considering KDTs even earlier. However, randomized clinical trials demonstrating efficacy in given diseases are available only for a few diseases. Moreover, to date, in Italy, except for GLUT1DS, we are still far from considering KDTs early in the therapeutic process. One of the main issues is that we do not have available biomarkers to predict KDTs' efficacy in terms of electroclinical parameters.

In the daily clinical practice of the consensus panel members, the ketogenic diet is proposed for 80–100% of patients with GLUT1 deficiency syndrome, 30–50% of patients with early infantile developmental and epileptic encephalopathy, 25–30% of patients with PDH deficiency and tuberous sclerosis complex. However, less than 10% of patients with malformations of cortical development and metabolic and mitochondrial diseases become KDT candidates.

**Figure 2:**
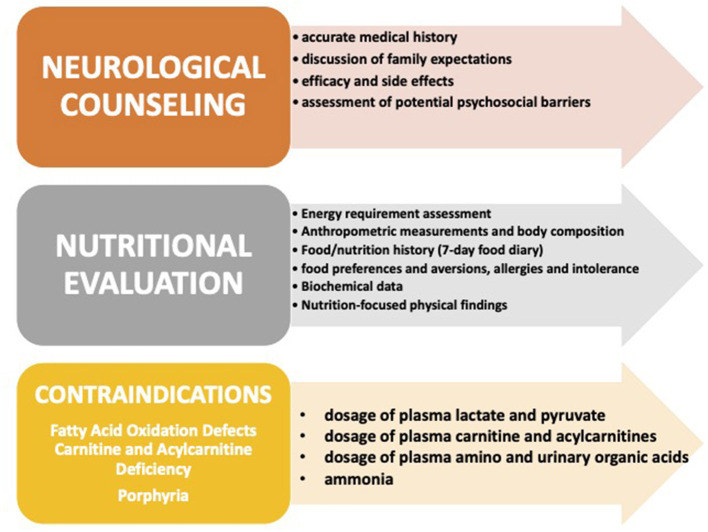
The pre-diet assessment.

A comprehensive and accurate evaluation of each patient before starting the diet will be crucial to understanding the real efficacy of the KDTs on each symptom in a single patient. This “pre-diet” evaluation should include a recollection of the electroclinical history of the patient to define epileptic syndrome and comorbidities and a re-evaluation of all the investigations performed to define the etiology. This reassessment aimed to investigate positive familial histories for hypercholesterolemia and/or metabolic diseases, with special attention to conditions that represent absolute contraindications to the use of KDTs (6) (see Figure 2). It is also fundamental to re-evaluate neuroradiological investigations to exclude symptomatic epilepsy eligible for the surgical approach, eventually repeating three Tesla magnetic resonance imaging (MRI), and to review the genetic investigations performed, with particular attention given to the results of the gene panel tests, to exclude patients for whom precision medicine is feasible. Moreover, genetic information might be crucial to interpreting treatment outcomes.

**Figure 3:**
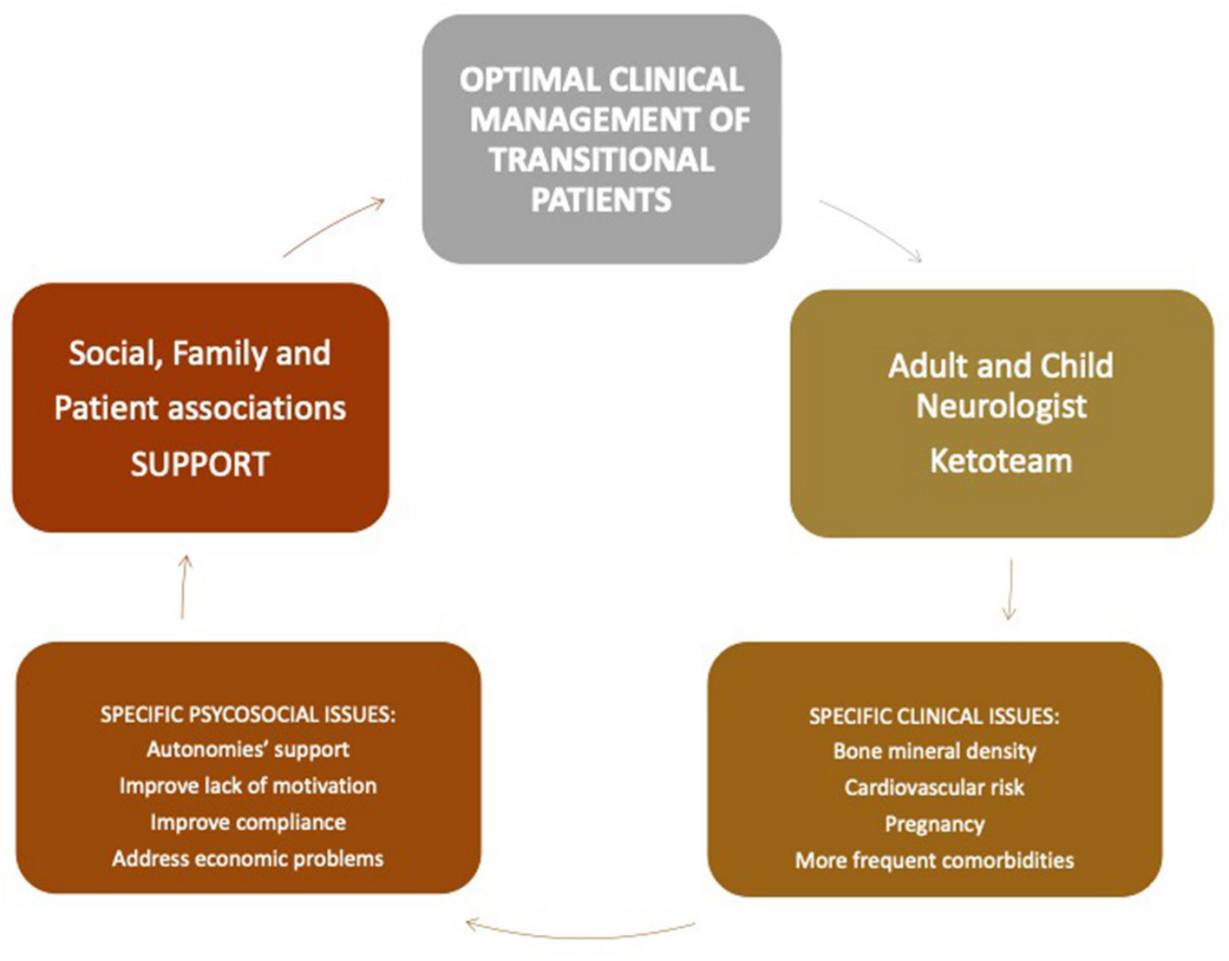
A follow-up proposal is provided, which should benefit from the transient coexistence of pediatric keto-team and adult neurologists in the identified national centers.

During the “pre-diet” assessment, the keto team should spend time with the family to discuss parental expectations and provide all the information necessary to initiate the diet. Indeed, a keto team is usually made up of neurologists, clinical nutritionists, and dieticians that work together with endocrinologists, psychologists, nurses, pediatricians, and family associations to provide adequate support to patients and caregivers from the very beginning (Figure 3). Besides, during this period, special help can come from families' associations through parent-to-parent support and from the use of videos, websites, and/or specific tools. For the clinical pre-diet assessment, refer to Figure 2.

## 4. Diet initiation

Flexibility and tailor-made protocols adapted to diet typologies and patient features, such as age, diagnosis, food acceptance, and socio-economic level, are strongly required in the KDT's initiation (8, 9).

Traditionally, KDT was started during hospitalization, after 24–48 hours of fasting, followed by the introduction of high-fat, adequate-protein, and low-carbohydrate foods (10). To date, many clinical studies have shown no significant difference in seizure reduction (11, 12), time of onset, and levels of ketosis (13) between non-fasting and fasting protocols, and the gradual onset of KDT showed fewer side effects and a better tolerance as well (14). A reduction in high-glycemic carbohydrate foods could be helpful a few days before starting KDT to prepare the child for the dietary change. Moreover, no fluid restriction is recommended (6).

Panel members agree to start cKD as an outpatient in most cases and to consider inpatient induction in specific conditions such as status epilepticus (100%), infants (100%), if there is a need for intensive caregiver training (80%), or in the presence of clinical or psychiatric comorbidities (80%−70%). The keto team should be flexible in deciding whether to hospitalize the patient for diet initiation, and sometimes families and patients should be allowed to choose to either start the diet as an inpatient or outpatient, especially in adulthood. The approach should be targeted to a single case, considering the therapeutic path's engagement and psychological impact on the entire family.

Hospitalization at cKD initiation is a common practice to closely monitor serum glucose, levels of ketosis, and side effects; moreover, under these circumstances, there is an opportunity to provide intensive training for caregivers on managing KDT at home. For this reason, hospitalization might be scheduled for neonates, patients with several comorbidities, or families who need intensive training to ensure adequate compliance.

Currently, in our clinical practice, most of the panel members (62,5%) plan to gradually increase the ketogenic ratio from 1:1 up to the desired ratio (3: 1 or 4: 1), depending on the ketonemia, with full calories from the beginning. The second utilized scheme is mainly scheduled for inpatients and consists in starting with 1/3 calories and a ketogenic target ratio from day one, with subsequent adjustments on a 5-to-7-day basis up to desirable.

The induction of the MCT-KD protocol is usually carried out at home without the need for hospitalization. Initially, small amounts of MCT are prescribed (e.g., 8–10 g per day), divided equally between the meals provided throughout the day, and/or partially substituted for the amount of fat already present in the diet. The proportion of MCT is then gradually increased until the desired percentage is reached (10–25% in children < 2 years, or 40–50% >2 years). This induction protocol limits the onset of gastrointestinal disorders, the typical side effects of this diet. However, MAD does not include a specific induction protocol, but a reduction of carbohydrate intake to 10–20 g per day is scheduled. MAD and LGIT are typically started in outpatient settings (6).

## 5. KDT and concomitant medications

KDT is usually added to previously administered ASMs. Despite the long history of concomitant use of the KDT and ASMs, their pharmacokinetic interactions remain unclear, and the literature in the field is still sparse and inconclusive. In addition, different results have emerged for pediatric populations compared to adult population studies.

Available data on pediatric populations show controversial results, ranging from the absence of relevant changes in anti-seizure drugs' blood concentration (15) to subtle changes in topiramate, levetiracetam, phenobarbital, and carbamazepine and possibly significant changes in valproate concentration under the influence of KDT (16).

Different results have been obtained in the adult population (17), where a drop of 10% in the mean concentrations of ASMs was observed on the MAD. After 4 and 12 weeks of MAD, drugs with higher serum concentration reductions were carbamazepine, clobazam (larger reduction), and valproate. However, levetiracetam appeared to be unchanged or even slightly increased. Moreover, a negative correlation was observed between changes in serum concentrations of drugs and urine ketosis.

Regarding the content of carbohydrates often found in liquid (syrup) formulations, the authors considered it more suitable, when feasible, to use syrups in other presentations without carbohydrates.

Approximately 75% of panel members did not find a significant modification of the ASM dosage following the introduction of KDT and, therefore, the need for dosage adjustments (for details, see Supplementary Table 1). Regarding the management of concomitant therapy with ASMs, the panel members recommend initiating drug decalage 6 months from the beginning of KDT in cases of diet efficacy.

## 6. Supplementations

KDT is characterized by the limitation/exclusion of many food groups, such as fruit, vegetables, cereals, legumes, and milk, determining a micronutrient intake lower than the recommendations and the need for their supplementation. Deficiencies are more common in the high ratio of cKD (18) and for group B vitamins, folates, vitamin D, vitamin C, calcium, phosphorus, magnesium, copper, zinc, selenium, and molybdenum (18–20).

Given the variable content of calcium in multivitamins, it may be necessary to combine a calcium supplement while avoiding excessive supplementation, increasing hypercalciuria, and facilitating the formation of stones.

Many factors can negatively affect bone health and growth in KDT patients: the impact of ketosis on the bone mineral buffer system, the reduced activation of vitamin D in the kidney, the reduced levels of growth such as IGF-1, the prolonged use of ASMs, and the reduced mobilization (21). It is essential to always consider that vitamin D status might not only be influenced by KDTs alone but also by ASMs and other factors. Thus, vitamin D values should be maintained in an optimally high range.

The international recommendations on KDTs emphasize that the evidence for potassium citrate supplementation is grade III (6), although the daily dose is not indicated. During KDTs, an increased risk of the incidence of kidney stones is described hypothetically as being due to acidosis, which increases the reabsorption of citrate into the proximal renal tubule and bone demineralization (22, 23).

Patients taking carbonic anhydrase inhibitors have an increased risk of developing metabolic acidosis. Some studies have reported the efficacy of potassium citrate in doses of 2 mEq/kg/day up to a maximum of 60 mEq/day in preventing nephrolithiasis and hypercalciuria in KD patients (24–26). Panel members recommend prescribing prophylaxis with potassium citrate at a dose of 2 mEq/kg/day. Importantly, the quantity of potassium to be considered is the estimated dietary intake with the prescribed diet.

Diet is the main source of carnitine for humans, particularly meat, milk, and cheese. Their endogenous production provides ~25% of carnitine, and the renal reabsorption capacity of carnitine can be maximized in conditions of low intake, such as a vegan diet (27–29). Patients treated with valproic acid and other ASMs may develop carnitine deficiency (30). Carnitine dosing is not routinely performed by all centers (31), and few studies have documented a transient reduction in free carnitine values during the initial months of KDT without justifying it as a priori supplementation (32, 33). With regard to the daily clinical practice of the panel members, a total agreement was found on the supplementation of carnitine only in cases of documented deficiency. Eventually, clinical and lab investigations might be scheduled for possible supplementation in cases of fatigue, difficulties in maintaining adequate ketosis values, or the use of drugs that reduce the availability of free carnitine.

Fiber is a nutrient that can be deficient in KDTs, particularly if the consumption of nuts, seeds, and any dietary foods with added fiber is low. Supplementation should be evaluated in cases of intestinal disorders such as constipation.

The low amount of water-rich foods, such as fruits and vegetables, in KDTs, determines the low water content of dietary plans. Patients should be encouraged to drink adequate water and sugar-free beverages to reach their recommended water requirements according to age. The amount of water can include both low mineral content water for hydration and high mineral (calcium) content water to cover mineral requirements according to the prescription. MCT, or medium-chain triglycerides, are used as a major source of fats in the MCT-KD. Dietary sources of MCT are mainly coconut and palm kernel oils. Pure MCTs are commercially available as dietary foods for special medical purposes and can also be present in food supplements. They are composed especially of caprylic acid (C8) and capric acid (C10) and lower amounts of caproic acid (C6) and lauric acid (C12) (34). Owing to their peculiar digestion, absorption, and mitochondrial transport, MCTs can enhance the production of ketones. The MCT daily dose should be carefully calculated, adjusted to the dietary prescription, and gradually introduced to avoid gastrointestinal side effects.

In conclusion, although the reference intake levels for micronutrients are studied for a healthy population (LARN 2014), they remain the main guide for planning the vitamin and mineral intakes of KDTs. The supplements recommended by the study group are listed in Table 1.

**Table 1:** Recommendations for KDT supplementations.

**Most frequent supplementations**
Sugar-free multivitamin with minerals (including trace minerals, especially selenium)
Calcium and vitamin D (respecting the daily requirement)
**Supporting supplementations**
Potassium Citrate
Vitamine D
Sugar-free prebiotics, Laxatives: Dietary fibers, mineral oil, glycerin suppository
Carnitine (if deficient and/or symptomatic patient)
MCT oil or coconut oil (source of MCT)

## 7. Follow-up management

Follow-up management provides serial evaluations that can be carried out on an outpatient basis or, better, as a day hospital at 1, 3, 6, and 12 months, while prolonged hospitalization is indicated in specific, selected cases.

The following should be scheduled before diet initiation and then at least once per year (Table 2).

**Table 2:** Suggested follow-up of clinical and instrumental management.

	**Evaluation**	**Pre-diet**	**1 month**	**3 months**	**6 months**	12 months^*^
Lab	Minimal	Metabolic screening CBC with formula, glycemia, ketones, electrolytes, bicarbonate, albumin, totlal protein, liver function, renal function, Vit D, B12, folate, complete urinalysis		CBC with formula, glycemia, ketones, electrolytes, bicarbonate, albumin, totlal protein, liver function, renal function, Vit D, B12, folate, complete urinalysis	CBC with formula, glycemia, ketones, electrolytes, bicarbonate, albumin, totlal protein, liver function, renal function, Vit D, B12, folate, complete urinalysis	CBC with formula, glycemia, ketones, electrolytes, bicarbonate, albumin, totlal protein, liver function, renal function, Vit D, B12, folate, complete urinalysis
	Optimal	PT, PTT, GH, IGF1, TSH, ASMs dosage	CBC with formula, glycemia, ketones, electrolytes, bicarbonate, albumin, totlal protein, liver function, renal function, Vit D, B12, folate, complete urinalysis	PT, PTT, GH, IGF1, TSH, ASMs dosage	PT, PTT, GH, IGF1, TSH, ASMs dosage	PT, PTT, GH, IGF1, TSH, ASMs dosage
Clinical	Minimal	Neurological, Nutritional	Telemedicine evaluation	Neurological nutrition	Neurological, Nutritional Telemedicine evaluation^*^	Neurological, Nutritional
	Optimal	Endocrinological neuropsychological emotional and behavioral	Neurological, Nutritional		Neurological emotion and behavioral	Endocrinological neuropsychological emotional and behavioral
Instrumental	Minimal	EEG, abdomen echo		EEG		EEG, abdomen echo
	Optimal	Calorimetry TSA ECG Echocardiogram Bone mineralometry			EEG, calorimetry	Calorimetry TSA ECG Echocardiogram Bone mineralometry

^*^After the first year of follow-up on a semestral/annual basis.

A laboratory screening: complete blood count (CBC) with formula, electrolytes (sodium, potassium, chlorine, calcium, phosphorus, magnesium, zinc, and selenium), bicarbonate, total protein, liver function [alanine transaminase (ALT), aspartate aminotransferase (AST), gamma-glutamyl transferase (GGT), total and fractionated bilirubin], albumin, prothrombin time (PT), partial thromboplastin time (PTT), renal function (creatinine, urea, and uric acid), lipid profile (total cholesterol, HDL, LDL, triglycerides, and total lipids), Vitamin D, GH, IGF1, TSH, the pharmacological dosage of ASMs (if applicable), and complete urinalysis.

Clinical evaluations: neurological, nutritional, endocrinological (if indicated - see below), neuropsychological, emotional, and behavioral (if applicable) evaluations.

Instrumental evaluations (annually or more frequently if necessary): electroencephalogram, calorimetry, abdomen echo, supra-aortic trunk echo color Doppler ultrasound, ECG +/- echocardiogram, and cardiological visit, if indicated. Computerized bone mineralometry, however, is recommended every 2 years.

Telemedicine visits, which have been recently implemented in clinical practice for epilepsy management (35), should be considered a valuable option in the long term when a patient on KDTs lives far away from the KDT Center or whether it is not possible to provide onsite visits.

Our consensus group recommends considering telemedicine evaluation at 1 month to verify the correct management of KDT, and after the first year of KDT as an alternative option, with follow-up on a 6-month basis beyond yearly onsite follow-up. Capillary blood ketonemia levels are considered more accurate than urine measurements (ketonuria) to evaluate ketone levels. Consensus group members also recommend obtaining blood ketones levels and glycemia twice a day (morning and evening before meals) in the first weeks of diet implementation and then during routine KDT clinic visits and according to patients' needs, KDT efficacy, tolerability, and compliance during follow-up.

## 8. Diet and side effect management

In the initial months of therapy, various adjustments are generally required to adjust the ketogenic ratio, maintain therapeutic ketosis, and meet the patient's satisfaction. During the follow-up in developmental age, these adjustments are also to adapt the dietary prescription to the growing child's changing needs, maintain adequate compliance, and address intercurrent illness.

In this step, the constant presence of the dietitian is essential to support the family and guarantee adherence to the diet. However, accompanying the family to achieve the goal with correct and consistent expectations can be challenging, especially in the first few weeks of a diet. Often, to achieve this goal, a change in the ketogenic ratio or a switch to another dietary protocol might be needed. Another situation that requires fine-tuning is an adjustment of the energy of the diet following episodes of hunger in the child, difficulties in taking the correct quantities of fluids and vitamin-mineral supplements, or alterations in the child's intestinal habits or post-prandial disorders. KDTs' side effects must be carefully researched and monitored to ensure the best tolerability of this treatment over time. It is essential to underline that the risk of side effects is reduced if patient selection is correctly conducted, the diet is carefully followed, and accurate clinical monitoring is guaranteed. In a systematic review of prospective cKD and MCTKD studies, more than 40 categories of adverse effects were identified, with gastrointestinal (constipation and vomiting), high serum lipid profiles, renal/genitourinary, and skeletal challenges being the most common (36) and often reversible in the short term if the diet is correctly designed. The most common side effects to be considered according to the specific timing of follow-up are listed in Table 3. In the long term, abnormal bone metabolism, growth failure, and rarer events (coagulation disturbance, pruritus, liver disease, iron deficiency anemia, hypogammaglobulinemia, and increased Q-T interval) have to be excluded. Very few KD patients developed selenium-deficient cardiomyopathy (37–39), and others developed neutropenia and copper-deficiency anemia (40, 41), but few studies are available on this topic. See Table 3 for the management and prevention of possible side effects.

**Table 3:** Management and prevention of most common KDT side effects.

**Side effect**	**Monitoring**	**Prevention**	**Management**
**0–3 months**			
Hypoglycemia (< 45 mg/dl)	Blood glucose capillary monitoring	Avoid prolonged intervals of daytime fasting and periodically re-evaluate dietary amount of carbohydrates and fat	1/2 glass of fruit juice
Hyperketosis (>6–7 mmol/l)	Ketonemia capillary monitoring	Avoid prolonged intervals of daytime fasting and periodically re-evaluate dietary amount of carbohydrates and fat	1/2 glass of fruit juice
Dehydration	Diuresis	Ensure adequate hydration	Increase liquids or sugar free water gels
**3–12 months**			
Constipation	Bowel habits	Ensure adequate hydration and fiber intake	Mineral oil, glycerin suppository
GE reflux	Specific symptoms	Appropriate anti-reflux behaviors	Prokinetics and acid blockers
Vitamin-mineral deficiency	Blood monitoring	Multivitamin with minerals supplementations	Specific supplementations
Hyperlipidosis	Blood monitoring	Prefer vegetable fats	Replace saturated with unsaturated fats; ω-3 fatty acids of MCT supplementation, reduction of the ketogenic rario
Kidney stones	Diuresis and specific symptoms abdomen ultrasound	Ensure adequate hydration and potassium citrate	Urologic management
>**12 months**			
Abnormal bone metabolism	Vit D and calcium monitoring bone mineralometry	Vitamin D and calcium	Endocrinological management
Growth failure	Auxological and endocrinological monitoring	Adequate caloric intake	Endocrinological management

## 9. Endocrinological assessment

The periodic auxological evaluation of patients undergoing KDTs is essential, especially considering the potential negative effect on growth occurring in a subject undergoing KDTs (42). Panel members recommend an auxological evaluation at least once during KDTs' treatment. In particular, expert pediatricians are required to perform appropriate auxological, physical, and instrumental semeiotics. Growth is considered an important indicator of the physical, psychic, and social wellbeing of both individuals and the population. Beyond an accurate physical examination aimed at identifying at first any dysmorphic note and disharmonic form of short stature, the essential measures to be detected are represented by the length and head circumference (up to 2 years), height (from 2 years), body weight, and body mass index (BMI). A very useful element is the growth rate, which allows a dynamic evaluation of growth trends and is expressed in cm/year. In the case of short stature, an adequate annual growth rate points toward constitutional hypostaturality but a growth rate below the normal threshold indicates a stature deficit, whose causes must be carefully considered. The height should always be examined concerning the genetic target and the bone age, which reliably expresses an individual's real biological maturity. Laboratory tests are valid support for clinical data aimed at certifying the suitability of the nutritional profile during KDT and excluding, in cases of reduced growth rate, the simultaneous presence of organ diseases, intestinal malabsorption, thyroid disease, and other endocrinological disorders. Finally, in older children and prepubertal individuals, a correct auxological evaluation should include a careful examination of the timing of pubertal initiation and the speed of progression of developmental signs, with possible execution of lower abdomen ultrasound in girls and testicular ultrasound in boys.

## 10. Transition, adulthood, and KDTs in the long term

Dietary therapies are more challenging to continue in adulthood since an adult neurologist and dietitian familiar with the different types of KDT are required (43, 44).

As with all pathologies of neuropsychiatric interest, epileptic patients on KDTs require detailed transition plans of medical care from pediatric to adult age (44). In the literature, few studies explore how to optimally manage patients using KDTs as they reach adulthood (45, 46).

Special considerations for adults should include supporting autonomy, giving attention to bone mineral density and vitamin D supplementation, managing treatment during pregnancy, and providing attention to possible comorbidities. Frequent support from the keto team to gather knowledge, skills, and confidence in patients managing KDTs is crucial. In Italy, as in many other countries, a specific KDT transition program is not available; therefore, many adult patients continue their dietetic and neurologic follow-ups in pediatric centers.

Among the panel members, KDTs are proposed in the first instance in adulthood mainly to patients with GLUT1 deficiency (60–80%), then to DEE (25%), and to other diseases such as MCD, metabolic, and mitochondrial diseases (< 10%).

There is limited evidence yet for the effectiveness of the KDTs in managing seizures in adults with intractable epilepsy. In the meta-analysis by Liu et al. (46), including studies on adult patients with intractable epilepsy who underwent CKD, MAD, LGID, and LGIT, the potential efficacy of KDTs in adulthood is invalidated by the loss of follow-up, lack of motivation, and poor compliance, mainly due to the onset of side effects. In addition, the lack of expert referents and specialized centers further increases the difficulty of evaluating and managing these patients in the transition period from childhood and adolescence to adulthood (46).

The primary resistances to KDT implementation in adults found in the practice of panel members are patient refusal, inadequate compliance perspectives, management of difficulties by the keto-team, severity of the clinical care picture, a lack of efficacy data, and costs (for details, see Supplementary Table 1).

The key aspect to consider during the management of clinical follow-up in the transition from adolescence to adulthood is not only to ensure the same control of seizures but also to pursue the greatest tolerability of the dietary regimen to achieve optimal compliance.

## 11. Discontinuation

Reasons for KDT discontinuation may include a lack of response, side effects, the need for nutritional status optimization, and medical emergencies and illnesses other than epilepsy. However, there are no clear guidelines in this regard, and the decision-making process should consider epilepsy etiology, individual response, tolerability, patient and family counseling, and EEG assessment before discontinuation.

KDTs should be used for at least 3 months to properly evaluate their efficacy (6). In this regard, KDT discontinuation should be considered after 3 months if unsuccessful or even before in cases of seizure worsening or side effects. However, KDTs may be continued for several years in patients achieving seizure freedom or nearly complete seizure control (e.g., >90% seizure reduction) with low side effects. Overall, in responder patients with at least >50% seizure reduction, KD discontinuation may be considered after ~2 years. However, there is no maximum duration. In specific conditions, the duration of the KD may be extended and even become lifelong, as in GLUT1DS, or shortened, as in infantile spasms and refractory status epilepticus (47).

Panel members recommend considering stopping KDTs in patients with seizure control of < 50% when compliance worsens, whether there is a specific request from the patient/caregivers and if the KDT duration is >2 years.

Detection of epileptiform discharges on EEG, malformations of cortical development or acquired brain abnormalities, the tuberous sclerosis complex, and a higher number of ASMs could represent predictive factors of seizure relapse to be considered in the decision-making process (48–51). Stepwise discontinuation is preferable, gradually changing ketogenic ratios over time and slowly adding carbohydrates. A slower weaning is preferred after a long time of KDT treatment, when KDTs show greater efficacy and when a higher number of ASMs are in use. The ratio can be reduced by 1:1 (e.g., from 4:1 to 3:1 to 2:1), associated with regular new carbohydrate food reintroduction while keeping calories constant and continuing nutrition supplementation over this period. If seizures relapse during or after discontinuation, KDTs can be increased to the previously effective ratio (6). A faster weaning might be chosen in the presence of a shorter KDT treatment duration and the presence of illness or poor compliance.

In the study by Worden et al. (49), no significant difference was detected in the incidence of seizure worsening between immediate (< 1 week), short (1–6 weeks), and slow (>6 weeks) rates of discontinuation. Overall, in patients at higher risk of seizure relapse, KD may be discontinued cautiously over 4–6 months.

For patients with low compliance as the main pivotal reason for discontinuation, transitioning from the ratios of classic KD to MAD or LGIT should be considered.

## 12. Effects beyond seizure

The achievable success of KD in seizure reduction does address a primary therapeutic concern. However, it is now widely shared that the efficacy of KDT might go beyond seizure reduction. Although not exclusively, GLUT1DS patients represent the most studied subjects undergoing KDT; these patients do have a striking and unsurprising benefit to seizures with KDT introduction, and thus, in the long term, there is more room for the evaluation of other disease domains. GLUT1DS patients often show a less severe overall clinical picture than other patients with DRE undergoing KDTs, who are often challenged by partial access to testing or are extremely far from the standard patients for whom existing questionnaires and evaluation tools have been validated. Moreover, no specific standardized and repeatable assessment tools have been implemented to verify the non-seizure benefits of KDTs on other disease aspects such as sleep, attention, motor performance, and behavior.

The available data in the literature, also confirmed in our experience, show that most parents report improved development and cognitive functioning, mainly in terms of shared attention and social interaction, language, and behavior (51). In some patients with such a global functioning improvement, it has been previously documented that there is an improvement in the structure and quality of sleep (52).

While waiting for standardized and targeted tools to measure different disease outcomes other than seizures, clinicians should meet patients' and parents' needs to address themes affecting the quality of life, such as development, language, attention, behavioral regulation, and social functioning. The correct collection and interpretation of these data should lead to a better approach to parents' and patients' counseling.

## 13. An integrated multidisciplinary approach and dialogue with families and patient associations

The term “patient engagement” refers to “the process of building a strong connection between patients, families, caregivers, as well as health care providers to facilitate and support the active involvement of patients in their care, to enhance safety and quality of health care service delivery” (53).

Much can be done to stimulate the “person-centered” virtuous process model regarding KDT management to ensure that patients adhere to dietetic therapies and maintain healthy behaviors to avoid new hospitalizations and improve their health care.

The most immediate solution is the establishment of an empathic relationship with the patient. There is a direct relationship between the clinician's compassion, the patient's engagement, and the subsequent commitment to improve health conditions and adhere to the diet. On this basis, a disease (and the diet to treat it) is not considered a biological event but something that affects the individual and affects their entire lives, both personally and relationally. Therefore, the clinician team that proposes a dietary approach to the patient should pay attention to the patient's needs. Moreover, the meaning (or non-meaning) that the patient attributes to the disease and dietary approach is relevant for an adequate therapeutic intervention, compliance, and the effectiveness of the treatment proposal. Therefore, a dialogue with the patient and the family is crucial; the representation of needs must be done by those who know their needs and the difficulties related to the disease. Good, simple, and clear communication between them is essential. If this occurs, it could significantly increase mutual understanding and give the patient and his family confidence. Finally, leveraging the outcome of this dialogue and one's own experience and skills is crucial for a dietician to build a diet that not only meets the identified nutritional parameters but also creates a palatable diet that complies with the family. It is essential to guarantee a multidisciplinary and holistic approach not only in the hospital: the patient and family need to feel that the clinician team will be present throughout the therapeutic diet journey, with the cooperation of Patients Associations as well, which can provide the support that often concerns not only the practical management of the diet, promoting “good practices” (e.g., giving some advice or suggesting tools useful in the management of the diet), but also all the bureaucratic issues regarding inclusion, health, and school management, and all the everyday life issues a family has to deal with during a KD plan.

## 14. Conclusion

These recommendations, developed within the Dietary Therapy Study Group of LICE, are an updated version of the previous Italian consensus of 2011 (54) on KDTs for epilepsy patients.

The aim of this study was to update them based on the most recent international literature data available and based on the international consensus (6), adapting them based on the social and dietary habits of the Italian population.

In clinical practice, KDTs might be highly effective in seizure control; the literature data of recent years proved a seizure frequency reduction of >50% in 35–56% of patients (55).

In the last few years, KDTs have become more widespread worldwide due to increasing knowledge and education, but the population of patients undergoing KDTs in Italy is smaller compared to other European and non-European countries. This might be because of deep-seated traditions that are difficult to overthrow, the complexity of follow-up and multidisciplinary management, and families' resistance. Besides, the high cost of prescriptions, only partially reimbursed by the National Health System, may also play a role. The proposed recommendations aimed to improve and facilitate the application of KDTs to expand their use, providing a tool based on the reality and needs of Italian clinicians and patients to promote local dissemination and implementation. The ultimate objective is not the mere overall spread of KDT utilization but the promotion of its good practice and optimal knowledge to achieve an effective and prompt offer of this treatment for the already shared epilepsy diagnoses and selected patients outcomes.

## Data availability statement

The original contributions presented in the study are included in the article/Supplementary material, further inquiries can be directed to the corresponding author.

## Author contributions

Conceptualization: VD, AT, LP, and PV. Methodology: VD, AT, FB, IB, AC, RC, FD, AD, ED, ML, SM, TM, FO, FR, ER, CV, LV, MZ, LP, and PV. Investigation and data curation: VD, AT, FB, IB, AC, RC, FD, AD, ED, ML, SM, TM, FO, FR, ER, CV, LV, MZ, LP, and PV. Writing—original draft preparation: VD, AT, FB, IB, AC, RC, FD, AD, ED, ML, SM, TM, FO, FR, ER, CV, LV, MZ, LP, and PV. Writing—review and editing: VD, AT, AD, LP, and PV. Project administration: VD. All authors have read and agreed to the published version of the manuscript.
