# Supplementary material for: Ketogenic dietary therapies in epilepsy: recommendations of the Italian League against Epilepsy Dietary Therapy Study Group
Source: Front Neurol. 2023 Jul 10;14:1215618. doi: 10.3389/fneur.2023.1215618 (PMC10368245; doi:10.3389/fneur.2023.1215618)
Supplement: Supplementary file 1 [file Data_Sheet_1.PDF]

QUESTION	OPTIONS	% of patients
Which *patients* in your daily clinical practice are most often offered the diet?	GLUT1DS	80-100%
	Developmental Epileptic Encephalopathies	30-50%
	PDH	25-50%
	Tuberous Sclerosis	25-30%
	Malformations of cortical development	25-30%
	Mitochondrial Diseases	20-40%
	Metabolic Diseases	5-10%
	Spasms	2-5%
	Other epilepsies	2-3%
Which patients do you propose *to continue* KDT in adulthood?	GLUT1DS	50-60%
	Malformations of cortical development	10-25%
	Developmental Epileptic Encephalopathies	5-10%
	Mitochondrial Diseases	2-5%
	Metabolic Diseases	2-5%
	Other epilepsies	2-5%
Which patients do you propose *to initiate* KDT also in adulthood?	GLUT1DS	60-70%
	Developmental Epileptic Encephalopathies	25%
	Malformations of cortical development	5-10%
	Other epilepsies	1-2%
	Other diseases (migraine, tumor, etc)	20%
What do you think is the bigger obstacle to starting KDT in adulthood?	Patient / caregiver resistance	70-100%
	Perspective of inadequate compliance	60-80%
	Difficult Ketoteam management	25-30%
	Clinical Severity	5-10%
	Lack of efficacy data	5-10%
	Costs	2-5%
What indicators do you use to evaluate whether to stop the diet when the efficacy on seizure control is <50%?	Compliance worsening	70-80%
	Patient / caregiver request	60-75%
	Need to increase /add ASMs	50-75%
	KDTs duration >2 years	30-50%
	EEG worsening	5-10%
In which other symptoms besides the seizure frequency have you found a benefit after KDTs introduction?	Attention	70-90%
	Social Functioning	50-60%
	Language	30-50%
	Behavior	30-40%
	Sleep	25-30%
	Motor Performances	5-50%* GLUT1
Do you find a growth deficit in your KDT patients on chronic therapy (> 1 year) ?	Yes	25%

QUESTION	OPTION	% OF CLINICIANS
What are the types of KDTs you use more frequently?	C-KD<br>MAD<br>MCT-KD<br>LGIT	65%<br>20%<br>10%<br>5%
Which type of C-KD induction do you practice?	Inpatient induction: 1/3 calories and target ketogenic ratio from day one with subsequent adjustments<br>Outpatient* gradual increase of the ketogenic ratio from 1:1 up to the desired ratio (3: 1 or 4: 1) depending on the ketonenia, full calories from the beginning<br>* Except for specific conditions requiring hospitalization	37,5%<br>62,5%
In patients candidate to KDTs, in what clinical conditions do you apply hospital induction?	Status epilepticus<br>Infants<br>Need for caregiver intensive training<br>Clinical comorbidities<br>Psychiatric comorbidities<br>Always<br>Never	100%<br>100%<br>80%<br>80%<br>70%<br>12,5%<br>12,5%
How quickly do you expect discontinuation in a patient in stable clinical condition when KDT is ineffective?	5-10 days<br>2-3 weeks<br>1-3 months	25%<br>50%<br>25%
In your clinical practice, do you plan to carry out an auxological evaluation at least once during a KDT treatment?	Yes<br>No	100%<br>0%
How often do you plan a bone densitometry in a patient in chronic KDT?	Biennal<br>Yearly<br>Never	12,5%<br>50%<br>37,5%
In which AEDs did you find a change in plasma dosage after the KDT introduction?	VPA<br>VPA (but not clinically significant)<br>PB – PHT<br>Never	12,5%<br>25%<br>12,5%<br>50%
After how long, in case of effectiveness of KDT, do you start the ASM decalage?	3 months<br>6 months	25%<br>75%
Which criterion do you choose for carnitine implementation	Low blood carnitine values<br>Low blood ketone values despite good adherence to KDT<br>Presence of drugs that can interfere with the bioavailability of carnitine (eg. VPA)	33,3%<br>33,3%<br>33,3%
Is there an adult transition program for patients treated with KDTs in your Hospital?	Yes<br>No	37,5%<br>62,5%
